# A Preliminary Investigation into the Use of Edge Illumination X-ray Phase Contrast Micro-CT for Preclinical Imaging

**DOI:** 10.1007/s11307-019-01396-5

**Published:** 2019-06-27

**Authors:** Charlotte K. Hagen, Marco Endrizzi, Rebecca Towns, Jeffrey A. Meganck, Alessandro Olivo

**Affiliations:** 1 Department of Medical Physics and Biomedical Engineering, University College London, Gower Street, London, WC1E 6BT UK; 2 Biological Services, University College London, Gower Street, London, WC1E 6BT UK; 3 Research and Development, Life Sciences Technology, PerkinElmer, 68 Elm St, Hopkinton, MA 01748 USA

**Keywords:** Micro-computed tomography, X-ray phase contrast imaging, Peclinical imaging

## Abstract

**Purpose:**

To enable a preliminary assessment of the suitability of edge illumination (EI) x-ray phase contrast (XPC) micro x-ray computed tomography (micro-CT) to preclinical imaging. Specifically, to understand how different acquisition schemes and their combination with dedicated data processing affect contrast-to-noise ratio (CNR) and spatial resolution, while providing control over scan time and radiation dose delivery.

**Procedures:**

Deceased mice (*n* = 3) were scanned with an EI XPC micro-CT setup operated under different settings, leading to scan times between 18 h and 13 min. For the shortest scan, the entrance dose was measured with a calibrated PTW 23344 ion chamber. Different data processing methods were applied, retrieving either separate attenuation and phase images, or hybrid (combined attenuation and phase) images. A quantitative comparison was performed based on CNR and spatial resolution measurements for a soft tissue interface.

**Results:**

All phase-based images have led to a higher CNR for the considered soft tissue interface than the attenuation image, independent of scan time. The best relative CNR (a sixfold increase) was observed in one of the hybrid images. Spatial resolution was found to be connected to scan time, with a resolution of approximately 20 μm and 60 μm achieved for the longest and shortest scans, respectively. An entrance dose of approximately 300 mGy was estimated for the scan performed within 13 min.

**Conclusions:**

Despite their preliminary nature, our results suggest that EI XPC bears potential for enhancing the utility of preclinical micro-CT, and, pending further research and development, could ultimately become a valuable technique in this field.

## Introduction

X-ray micro-computed tomography (micro-CT) has gained major significance in the area of preclinical imaging of small animals, due to its ability to provide spatial resolutions on the micrometre scale and within relatively short scan times. However, this modality is affected by one limiting aspect, namely a relatively poor ability to visualise soft tissues. This is due to the fact that image contrast is generated from x-ray attenuation; while this leads to a strong contrast between tissues with strong attenuation differences (*e.g.,* bone, lung parenchyma and surrounding structures), it proves to be suboptimal for visualising the structures with weak or similar attenuation (such as soft tissue). This could be improved by selecting a higher tube current or extending the exposure; both would reduce noise and, hence, increase contrast-to-noise ratio (CNR). However, both would simultaneously increase the radiation dose, which may not be practicable when imaging live animals. An alternative approach is to use contrast agents, which can locally change the attenuation properties of tissue; however, contrast agents vary widely in their tissue specificity [1] and toxicity.

The development of x-ray phase contrast (XPC) micro-CT, which exploits phase effects for image formation, could provide a solution [2, 3]. This type of imaging focusses on the fact that x-rays are electromagnetic waves with an amplitude and a phase and takes into account the phase shift that occurs during interaction with matter, rather than, or in addition to the amplitude reduction caused by attenuation. The extent to which both effects are caused by a sample in the beam is described by the complex refractive index, *n* = 1−*δ* + *iβ*, which depends on the x-ray wavelength (*λ*). Its imaginary part (*β*) is related to the linear attenuation coefficient (*μ*) *via μ* = (4*π*/*λ*) × *β*, and the decrement from unity of its real part (*δ*) describes the phase shift. The fact that, within the diagnostic energy window (10–100 keV), differences in *δ* between weakly attenuating materials (such as soft tissue) can be up to three orders of magnitude larger than in *β* (see Table 1) is often considered the rationale behind XPC imaging, since, if properly exploited, this can lead to a significant increase in CNR.

**Table 1:** Decrement from unity (*δ*) of the real part and imaginary component (*β*) of the complex refractive index of different tissues at 18 keV, according to http://ts-imaging.science.unimelb.edu.au/Services/Simple/ [4]

Material	Bone	Muscle	Lung	Fat	Skin
*δ* (at 18 keV)	12.71 × 10^−6^	7.41 × 10^−7^	7.42 × 10^−7^	6.58 × 10^−7^	7.66 × 10^−7^
*β* (at 18 keV)	5.53 × 10^−7^	6.06 × 10^−10^	6.12 × 10^−10^	3.02 × 10^−10^	6.01 × 10^−10^

Several XPC techniques have been developed [5], which can be broadly categorised into free space propagation-based, crystal-based, interferometric and non-interferometric grating-based approaches. The fact that XPC imaging has been demonstrated to be feasible with laboratory-based (*i.e.,* non-synchrotron) sources [*e.g., *6–9] has important implications for use in small animal imaging; it potentially makes the technology more widely accessible, as relatively affordable and deployable scanners can be built. The first laboratory XPC micro-CT device targeted specifically at preclinical imaging was developed by Tapfer and colleagues at the Technical University of Munich [10], based on grating interferometry. Their system has been a pioneering development as it was (and still is) the first machine with a rotating gantry [11]. In contrast to non-gantry systems in which the sample is rotated, it allows keeping the animal stationary during scans, easing the use of anaesthetic equipment. That machine was used for dark field imaging of mouse lungs *ex vivo* [12] and *in vivo* [13], the results suggesting that the accessible small-angle scattering information can aid the diagnosis of pulmonary emphysema. This research was complemented by the development of XPC micro-CT systems based on free space propagation, which have provided improvements in tumour demarcation over purely attenuation-based scans [14], as well as the ability to image mouse lungs *in situ* with approximately 5 μm spatial resolution [15].

At UCL, we have carried out intensive research into developing an alternative XPC technique, called edge illumination (EI), with the aim of fulfilling the criteria of affordability and deployability (through a robust experimental setup). EI has been used previously in the context of small animal imaging for planar (2D) XPC imaging of rat articular cartilage *ex vivo*, demonstrating its ability to detect minute blemishes equivalent to those that could be an early indicator of osteoarthritis [16]. In this paper, we report on the first EI XPC micro-CT images of deceased mice, and evaluate, in a preliminary manner, its suitability to preclinical imaging. This study explores the feasibility of performing scans within appropriate time frames (short enough to keep animals anesthetised) and radiation doses (low enough to allow repeated imaging of the same animal), both of which are essential for a potential implementation of the technique in preclinical imaging. For this purpose, we evaluate how key image quality metrics (CNR, spatial resolution) in EI XPC micro-CT are affected by different combinations of acquisition schemes and processing methods, while aiming to minimise scan time and radiation dose.

## Methods

### Working Principle

**Fig. 1.**
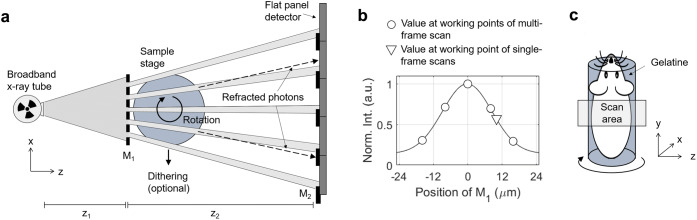
**a** Schematic of an EI XPC micro-CT setup (seen from top and not to scale). **b** Illumination curve obtained for the experimental setup with the values at the working points used in this study. **c** An illustration of the scanned samples.

EI originates from the idea to align a thin, laminar x-ray beam with the top edge of a row of detector pixels. When a sample is scanned through such a setup, the phase shift introduced by the sample causes a small angular displacement of the beam, a phenomenon known as refraction. The edge alignment allows translating refraction into intensity variations, as the angular beam displacement leads to the detection of a higher or lower number of photons, depending on the refraction direction. The adaptation of this concept to 2D flat panel detectors is illustrated in Fig. 1a. Instead of a laminar beam, the setup employs an array of narrow x-ray beamlets created by a mask (M_1_) upstream of the sample. A second mask (M_2_) in front of the detector creates sharp edges between the pixels. By adjusting the position of M_1_ (along the *x*-axis), the x-ray beamlets can be aligned with M_2_ in such a manner that edges and pixels are both illuminated by a fraction of each beamlet. In analogy to the original laminar beam setup, refraction can now be sensed for each beamlet.

In addition to refraction, EI is naturally sensitive to x-ray attenuation. The intensity in each raw projection image is a combination of both, and can be described by *I*(*x*, *y*) = *e*^−*μ*(*x*, *y*)^*C*(*x*_m_ + *α*(*x*, *y*)*z*_2_/mag), where *μ*(*x*, *y*) = 4*π*/*λ* ∫ *β*(*x*, *y*)*dz* is the sample’s projected linear attenuation coefficient, *α*(*x*, *y*) = (*λ*/2*π*) ∙ *d*/*dxΦ*(*x*, *y*) is the refraction angle, *Φ*(*x*, *y*) = 2*π*/*λ* ∫ *δ*(*x*, *y*)*dz* is the phase shift, *z*_2_ is the mask-to-mask distance, mag is the system magnification, and *x* and *y* are the horizontal and lateral detector coordinates. The symbol *C* denotes the illumination curve, which can be measured in the absence of the sample by step-scanning M_1_ along the *x*-axis and recording the intensity on the detector at every step (Fig. 1b). This curve, which is of approximately Gaussian shape when measured across a single period of M_1_, characterises the performance of any EI setup and should be acquired before each scan. The value *x*_m_, describing the position of M_1_ at which the image is taken, is the called the working point. In the raw data, the refraction contrast appears as an edge signal characterised by fringes at boundaries within the sample, which corresponds to the refraction angle being the differential of the phase shift.

Certain setup design criteria must be met in order to ensure the compatibility of the EI sensing mechanism with spatially incoherent or partially coherent radiation (*i.e.,* extended source focal spots). Specifically, the period of M_1_ must be sufficiently large to ensure that any overlap of the beamlets, where present, is only due to the tails of the source distribution. Achromaticity, meaning that the imaging system does not weigh energies differently from each other, has been demonstrated [17]. Any energy dependence of image contrast is therefore only due to the sample material itself (due to the wavelength dependency of the complex refractive index). Hence, the broadband energy spectra of laboratory x-ray sources are being fully exploited.

Further, EI provides flexibility in terms of the physical quantities that can be reconstructed into tomographic images, enabled by the option to combine different acquisition schemes with dedicated phase retrieval methods. For example, separate attenuation and phase images can be retrieved, or, like in grating interferometry, a third, ultra-small-angle scattering channel can be accessed when the method is operated in dark field mode [18]. Alternatively, the refraction-based edge signal can be converted into area signal and merged with the attenuation contrast, enabling the reconstruction of hybrid (*i.e.,* combined phase and attenuation) images [19]. As a second flexible aspect, the spatial resolution can be adjusted *via* the acquisition parameters [20]. When the sample is simply rotated during a scan, the spatial resolution is essentially confined to the effective pixel size (“at-pixel resolution”). However, when performing dithering, a process by which the sample is also scanned along the *x*-axis in multiple sub-pixel steps per rotation angle, data are taken at each step and combined into a single projection that features a higher sampling rate, spatial resolutions below the effective pixel size (“sub-pixel resolution”) can be achieved without the need for any post-processing step such as deconvolution. This is a unique feature that is a consequence of structuring the beam into an array of beamlets.

### Acquisition Schemes and Data Processing

In this study, we have exploited this flexibility and applied three different acquisition schemes which were combined with dedicated data processing methods in order to explore how key image quality metrics (CNR, spatial resolution) are affected when the scan time is gradually reduced to a level that is appropriate for preclinical imaging. This has led to the following types of images: (i) separate attenuation and phase images with sub-pixel resolution, (ii) hybrid (*i.e.,* combined attenuation and phase) images with sub-pixel resolution and (iii) hybrid images with at-pixel resolution. Brief explanations of the applied acquisition schemes and processing methods are provided below. In addition, key parameters, including scan times and an estimate of the radiation dose delivered during the shortest scan, are summarised in Table 2.

**Table 2:** Parameters of the different EI XPC micro-CT acquisition schemes

Scanning scheme	Separate attenuation and phase images, sub-pixel resolution	Hybrid images, sub-pixel resolution	Hybrid images, at-pixel resolution	Hybrid images, at-pixel resolution (fast and low-dose mode)
Source filtration	30 μm Mo filter	None	None	30 μm Mo filter
Working points	5	1	1	1
Dithering steps	4	4	1	1
Angular views	1441 over 360 degrees (0.25 degree step)	721 over 360 degrees (0.5 degree step)	721 over 360 degrees (0.5 degree step)	721 over 360 degrees (0.5 degree step)
Exposure time per frame	1 s	4 s	4 s	1 s
Average dead time per frame (for read-out and motor movement)	Approximately 0.8 s	Negligible (ms)	Negligible (ms)	Negligible (ms)
Acquisition mode	Step-and-shoot	Continuous (4 scans, one per dithering step)	Continuous	Continuous
Reference images	5 per angular view	10 before and after each of the 4 scans	10 before and after the scan	10 before and after the scan
Total scan time	Approximately 18 h	Approximately 3 h 20 min	51 min	13 min
Entrance dose	Not measured	Not measured	Not measured	Approximately 300 mGy

### Separate Attenuation and Phase Images with Sub-pixel Resolution

The reconstruction of these images was enabled by a multi-frame phase retrieval method [21], which requires that, at each rotation angle, frames are collected at several working points. Data were taken at five working points, *x*_M_ = −16, 8, 0, 8 and 16 μm, corresponding to the tails, mid-slopes and top of the illumination curve (Fig. 1b). The retrieval method, which involves fitting a Gaussian function to the acquired frames on a pixel-by-pixel basis, extracts the refraction angle for each beamlet, as well as the attenuation. Dithering was performed in order to increase the spatial resolution to below the effective pixel size, which involved step-scanning the sample four times by a distance of 12 μm (corresponding to a fourth of the period of M_1_). The tomographic scan involved rotating the sample in angular increments of 0.25 degrees over 360 degrees, and the frames at the different dithering positions and working points were collected for each angle. Tomographic reconstruction was performed using filtered back projection. In the case of the phase data, this required applying a special filter function (Hilbert filter) to accommodate the differential nature of the refraction signal. A sample “jitter” was applied to reduce ring artefacts; this involved translating the sample along the x-direction by a random integer multiple of the effective pixel size at each rotation angle and (digitally) reversing this shift sequence when processing the data. The scan was performed in step-and-shoot mode, meaning that the sample was kept in a fixed position while the detector was acquiring, and moved (*i.e.,* translated, rotated) in between the acquisition of frames. The necessity to acquire data at several working points imposes dead times in between the acquisition of the individual frames in order to shift M_1_ to the next position, which increases the overall duration of the scan.

### Hybrid Images with Sub-pixel Resolution

The reconstruction of hybrid images, in which attenuation and phase contrast are combined, was enabled by a single-frame phase retrieval method [19]. This requires that frames are collected at only one working point per rotation angle. In our scan, this was *x*_m_ = 10 μm, corresponding to the right mid-slope of the illumination curve (Fig. 1b). The retrieval involves applying a low-pass filter to each frame, which converts the refraction-based edge signal into area signal and merges it with the attenuation signal. The method relies on the assumption that *δ* and *β* are proportional across the sample and that the proportionality factor is known, which is used to calculate the filter. If this assumption is violated, the method only allows accurately retrieving the interfaces between any two materials in the sample; interfaces between other materials are either “under-retrieved”, leading to a residue of edge contrast, or “over-retrieved”, causing blurring. Again, four dithering steps (12 μm each) were applied to achieve sub-pixel resolution. However, this time, we have completed four individual scans in continuous mode (the sample was rotated without interruption), with the sample being moved to the next dithering position in between these scans. Each of the four scans involved rotating the sample over 360 degrees and acquiring frames for each 0.5 degree increment. The sinograms obtained from the individual scans were combined, and tomographic images were reconstructed using filtered back projection. No specialised filter function is required for the hybrid reconstruction, as the differential refraction signal is converted into area signal already during phase retrieval. A ring removal technique, based on median filtering the tomographic images in polar coordinates and subtracting the filtered from the original data, was applied post reconstruction. To provide alternative display options, maximum and minimum intensity projections were computed from the reconstructed stack of axial tomographic slices by considering 35 adjacent slices, which corresponds to a vertical distance of approximately 1.6 mm.

The continuous scanning mode, enabled by the need for only one working point per rotation angle and dithering step, together with the increase of the angular sampling interval, has led to a substantial reduction in scan time compared with the previous step-and-shoot scan (3 h 20 min instead of 18 h, see Table 2), as lengthy dead times required for the repeated repositioning of M_1_ were eliminated.

### Hybrid Images with At-pixel Resolution

Next, we performed a single-frame scan without dithering, leading to at-pixel (instead of sub-pixel) resolution. Again, the working point at which all frames were acquired was *x*_m_ = 10 μm; the tomographic scan involved rotating the sample continuously over 360 degrees and acquiring frames for each 0.5 degree increment. Filtered back projection was used for tomographic reconstruction, and the same ring removal technique as above was applied. These parameters have led to another fourfold reduction of scan time compared with the previous scan (from 3 h 20 min to 51 min, see Table 2). Besides reconstructing a stack of axial slices, the dataset was also visualised as a 3D rendering using the open-source software platform 3D Slicer [22].

Finally, we repeated this scan while reducing the exposure time by a factor of four (from 4 s to 1 s per frame), leading to a total scan time of 13 min. We have also placed a 30-μm molybdenum (Mo) filter in front of the source, so as to attenuate the low end of the polychromatic spectrum, reducing the dose delivery. The entrance dose for this last scan was measured with a calibrated PTW 23344 ion chamber placed at the sample position. The ion chamber was certified to an uncertainty 3.3 %, which is composed of the uncertainties of the calibration procedure and those of the specimen during calibration. The chamber reading was approximately 0.4 mGy per s, which corresponds to an entrance dose of around 300 mGy when extrapolated to the entire scan.

### Experimental Setup

The experimental setup featured a rotating Mo anode x-ray tube (MicroMax-007 HF, Rigaku, Japan), operated at 40 kV and 30 mA. These settings correspond to a focal spot of approximately 70 μm horizontally and a broadband spectrum with a mean energy of approximately 18 keV. The detector was a photon-counter (Pixirad-2) with a pixel size of 62 μm. The distance between the source and M_1_ was 1.6 m (z_1_), and that between M_1_ and M_2_ was 0.4 m (z_2_) (corresponding to a magnification of mag = 1.25). The period of M_1_ was 48 μm, and its aperture width was 12 μm. Mask M_2_ had a period and apertures of 59 μm and 15 μm, respectively. Both masks were manufactured by electroplating gold onto a graphite substrate, resulting in a periodic array of absorbing (gold) and transmitting (graphite) columns.

### Sample Preparation

The scanned animals (mice, Charles River, *n* = 3) were sacrificed at 10, 15 and 18 days of age in accordance with Schedule One of The Animals (Scientific Procedures) Act 1986 amendment regulations 2012. Within approximately 1 h euthanasia without any other preparation, they were placed in plastic cylinders of 1.9 cm in diameter (Fig. 1c). Any remaining empty space was filled by injecting gelatine (agarose gel 3 % approximately, UltraPure TM agarose from Invitrogen) into the cylinders, which solidified when cooling down and ensured that motion artefacts were minimised. Despite our best efforts to remove air pockets from the gelatine, some small air bubbles and a layer of air between the animals and the cylinder remained present. The relatively small effective vertical field of view of the Pixirad detector (approximately 2 cm) has restricted our scans to only a part of the animals. In line with previous studies in which the suitability of XPC micro-CT for preclinical imaging was investigated [12, 13, 15], we focussed on scanning the chest area (including the lungs). Although we were also interested in visualising the abdomen, this was not feasible due to air bubbles trapped in the intestinal tract. These moved and expanded during scans, causing strong motion artefacts which made an effective image reconstruction impossible.

## Results

**Fig. 2.**
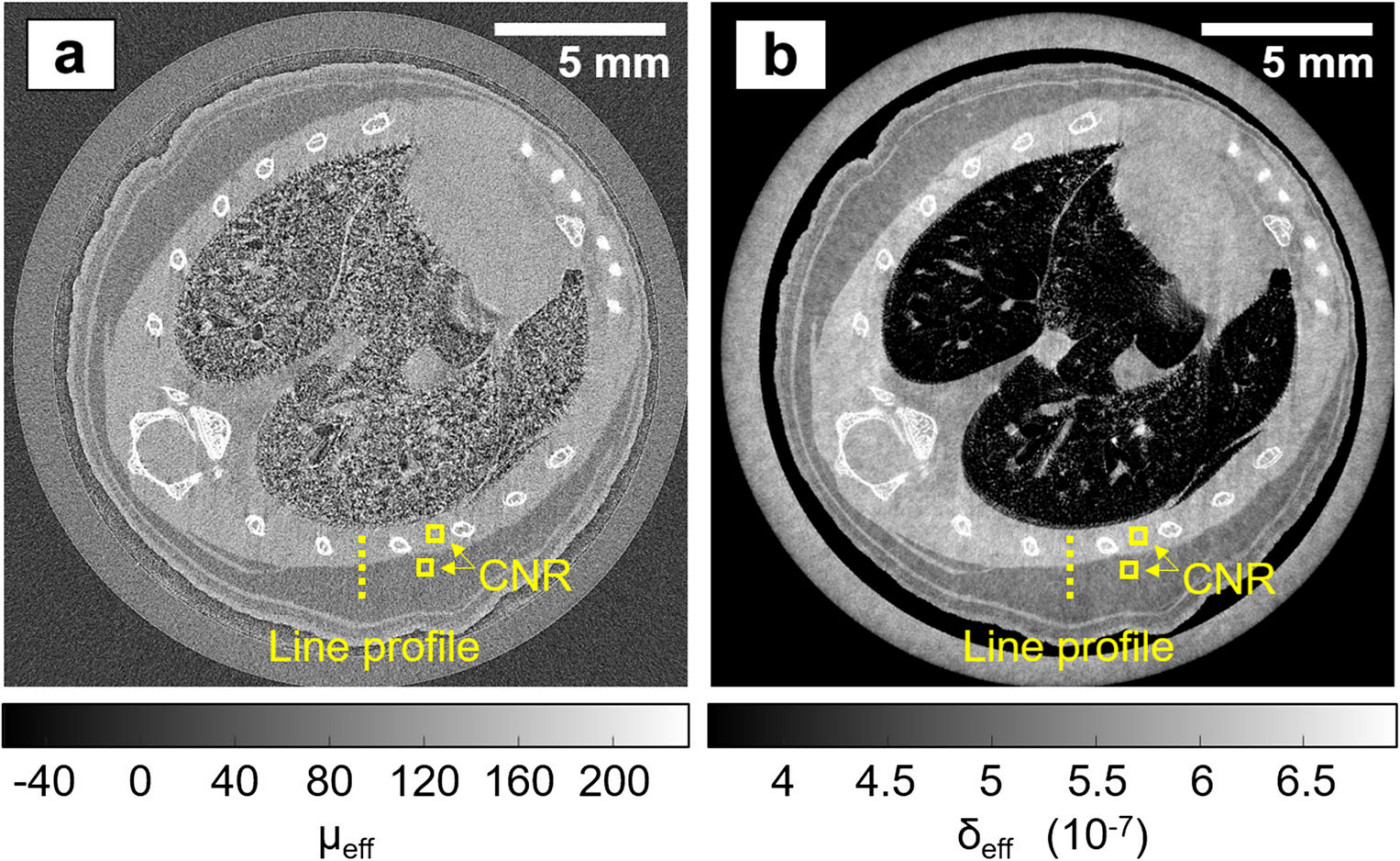
Sub-pixel resolution EI XPC micro-CT images of a mouse chest, acquired with the dithered, multi-frame scheme. **a** Reconstructed attenuation image. **b** Reconstructed phase image.

**Fig. 3.**
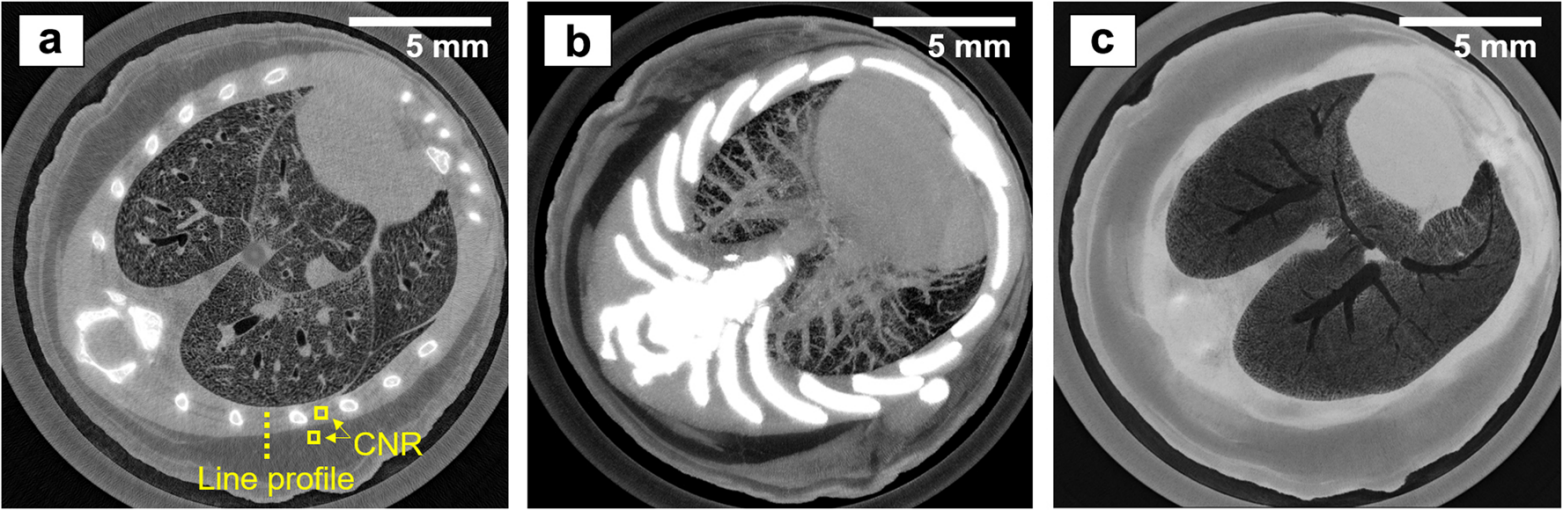
Sub-pixel resolution EI XPC micro-CT images of a mouse chest, acquired with the dithered, single-frame scheme. **a** Hybrid reconstruction showing a combination of phase and attenuation contrast. **b** Minimum intensity projection over part of the reconstruction volume. **c** Maximum intensity projection over part of the reconstruction volume.

**Fig. 4.**
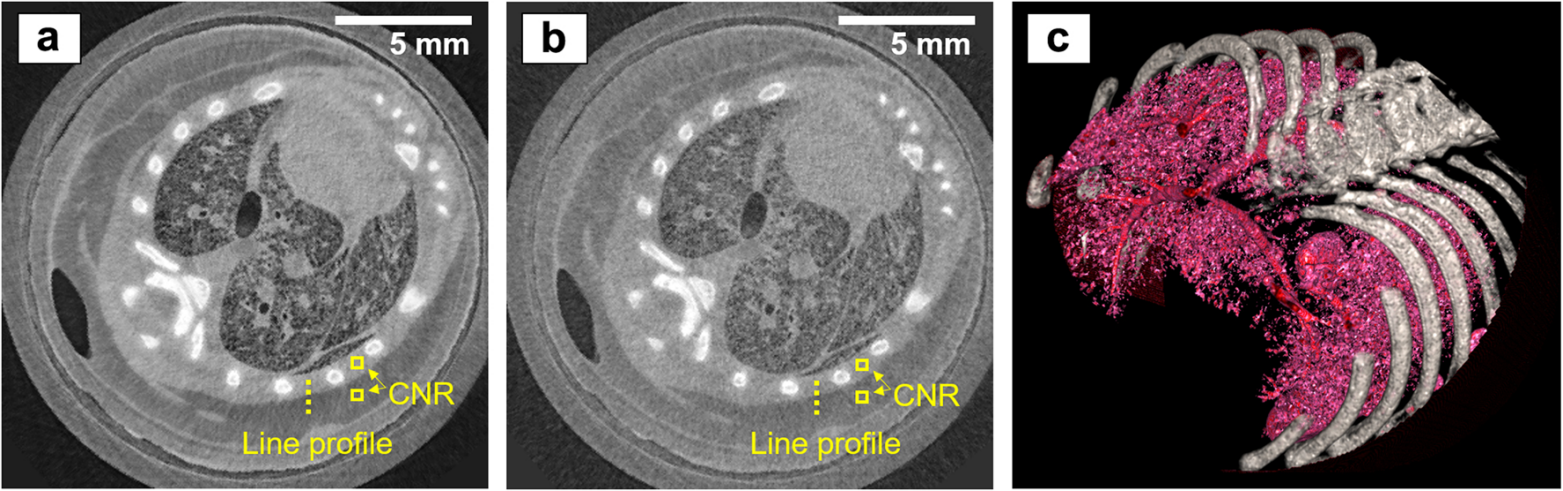
At-pixel resolution EI XPC micro-CT images of a mouse chest, acquired with the non-dithered, single-frame scheme. **a** Hybrid reconstruction showing a combination of phase and attenuation contrast. **b** Low-dose hybrid reconstruction (acquired with a 30 μm Mo filter). **c** 3D rendering of the rib cage and lung structures based on the data shown in **a**.

The separate attenuation and phase images with sub-pixel resolution, reconstructed from the dithered multi-frame scan, are shown in Fig. 2; the hybrid images with sub-pixel resolution, obtained from the dithered single-frame scan, are shown in Fig. 3, and the hybrid images obtained from the much faster, non-dithered single-frame scans, including the 13 min acquisition, are shown in Fig. 4. An axial slice through the animals’ chest is presented for each scan. The attenuation and phase images show maps of the effective linear attenuation coefficient (*μ*_eff_) and *δ*-value (*δ*_eff_) of the different tissues. The hybrid images are not generally quantitative due to the approximations made in the phase retrieval. Display levels/windows have been chosen such that the grey values for fat are roughly comparable in all images (note that this has led to a “disappearance” of the lung structure in the phase image). Alternative display options (minimum and maximum intensity projections over a part of the reconstructed volume, a 3D rendering) are provided for a subset of scans. While the axial slices have the advantage that they remove any overlap between structures located at different vertical depths, minimum and maximum intensity projections can provide a better visualisation of specific features, such as the rib cage or the lung vascular tree. The 3D rendering allows understanding the reconstructed features in their anatomical context.

**Fig. 5.**
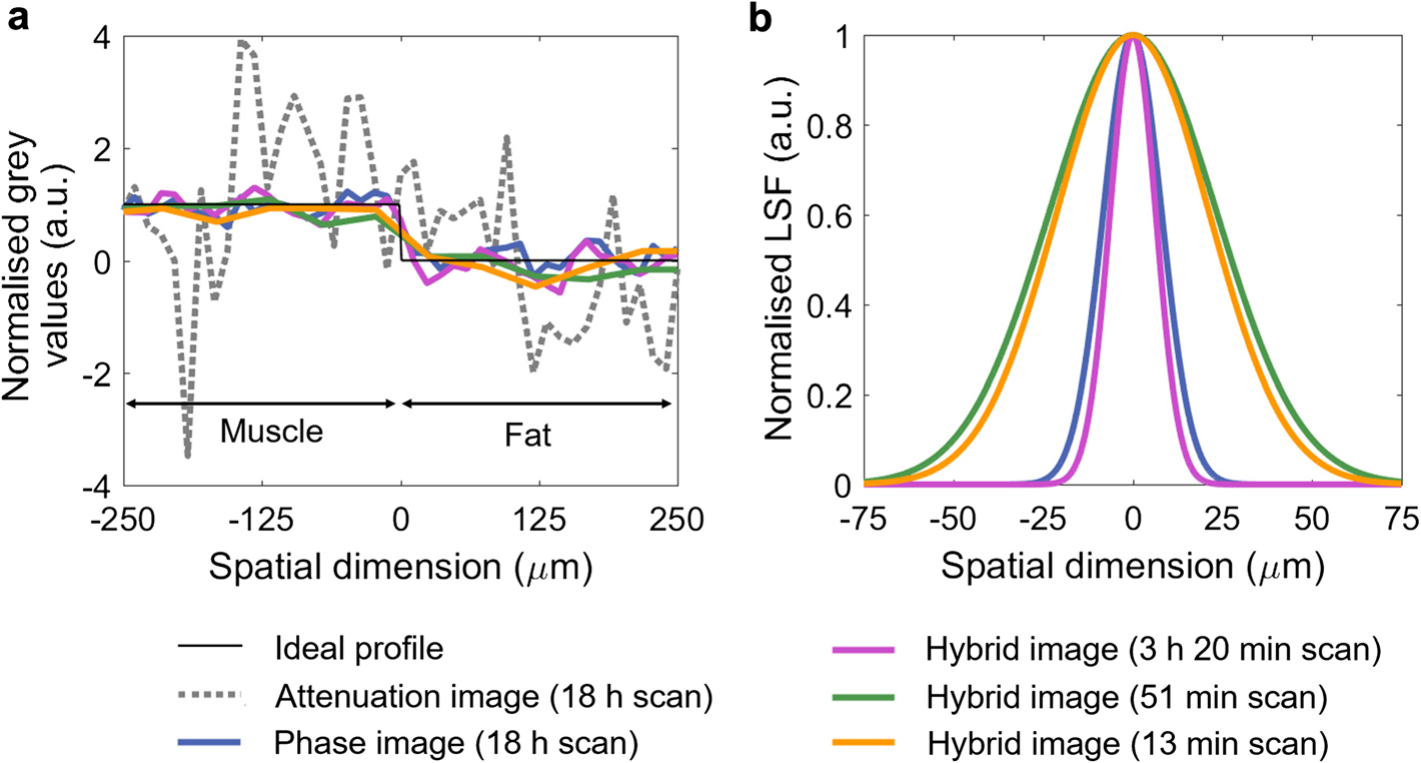
**a** Line profiles across the muscle-fat interface extracted from the sub-pixel resolution attenuation and phase images, and the sub-pixel and at-pixel resolution hybrid images. **b** Line spread functions derived from these profiles.

A comparison of the axial slices reveals that bones appear with a much stronger contrast (relative to the surrounding chest muscle tissue) in the attenuation image than in the phase image, while they appear blurred in all hybrid images. The strong attenuation contrast reflects one of the strengths of attenuation-based micro-CT (excellent visualisation of hard, calcified tissue). The blur in the hybrid images is due to the fact that the presence of bones severely violates the assumption underpinning the single-frame phase retrieval (*i.e.,* that *δ* ∝ *β* across the sample), as the relationship between the *δ* and *β* of bone is fundamentally different of the relationship between the *δ* and *β* of soft tissue (Table 1). It can further be observed that, contrary to the bones, soft tissue structures in the attenuation image are less pronounced and appear grainier than in the phase and hybrid images. This is also shown by the line profiles plotted in Fig. 5a, which were extracted across the interface between the chest muscle and subcutaneous fat layer, as indicated by the dashed line in the images. Indeed, the attenuation profile is corrupted by noise, to an extent that it is difficult to identify the muscle-fat separation. In comparison, the phase and hybrid profiles are much smoother and clearly reveal the interface between the two tissues.

To quantify these observations, the CNR, defined as |(*S*_1_−*S*_2_)|/*σ*_2_, where *S*_1_ and *S*_2_ are the mean grey values reconstructed for muscle and fat, respectively, and *σ*_2_ is the standard deviation for fat, was calculated for the squares shown in the respective figures. For the sub-pixel resolution images, each square measured 30 by 30 pixels, while for the at-pixel resolution images, each square measured 10 by 10 pixels. The results are listed in Table 3. While the attenuation CNR might be lower than values reported elsewhere [*e.g., *23], it is expected that CNR values differ for different experimental conditions. The values reported here serve the purpose of comparing images acquired with the same setup, though it should be noted that retrieved attenuation images may not be representative of conventional micro-CT images generally due to the fact that a fraction of each beamlet is absorbed by M_2_. Nevertheless, the comparatively high CNR in the phase and all hybrid images, including for the shortest scan performed in 13 min, demonstrates the strength of XPC techniques for visualising soft tissue interfaces. The CNR in the hybrid images exceeding that in the phase image (apart from the fastest and low-dose case, which is discussed below) is due to the hybrid approach combining contrast contributions from both the phase and the attenuation channels. When comparing the different hybrid images, it can be noted that no loss of CNR was suffered when reducing the scan time from 3 h 20 min to 51 min. This is because, in the shorter scan, less dithering steps were acquired, which reduces spatial resolution, but does not affect the photon statistics per image pixel. In fact, the CNR obtained from the shorter of the two scans is even slightly higher, although this might be an arbitrary fluctuation and should not be considered indicative. On the other hand, a drop in CNR can be observed when reducing the scan time further to the shortest value of 13 min. Here, the application of the beam filter (30 μm Mo) has led to a reduction of the intrinsic contrast between muscle and fat, as a consequence of attenuating the low energy part of the spectrum.

**Table 3:** Summary of the CNR and spatial resolution measurements in the different reconstructed images

Reconstructed image	Attenuation with sub-pixel resolution (18 h)	Phase with sub-pixel resolution (18 h)	Hybrid with sub-pixel resolution (3 h 20 min)	Hybrid with at-pixel resolution (51 min)	Hybrid with at-pixel resolution (13 min)
CNR	0.9	3.1	5.0	5.4	3.1
Spatial resolution (FWHM)	Not measured	19 μm	15 μm	55 μm	50 μm

Besides CNR, another metric that influences the visualisation of tissue structures is spatial resolution. As per choice of the scan parameters (Table 2), the different acquisition schemes have led to different spatial resolutions. While theoretically this metric is, to first approximation, defined by either the dithering step, or, if no dithering is applied, by the period of M_1_, in practice, it is influenced by additional factors such as, *e.g.,* blurred edges due to unavoidable sample motion artefacts or system instabilities. To estimate the spatial resolution in the reconstructed images, error functions were fitted to the muscle-fat profiles, their derivatives were calculated to obtain line spread functions (LSF), and full width at half maxima (FWHM) were extracted. The LSF plots are shown in Fig. 5b, and the corresponding spatial resolution estimates are listed in Table 3. Note that it was not possible to analyse the attenuation profile with a reasonable degree of accuracy due to the high level of noise. All resolution estimates are indeed close to the expected values; in the dithered images, we have measured 15 and 19 μm, respectively, while the expected value would be 12 μm (*i.e.,* the dithering step), and in the non-dithered images, we have measured 55 and 50 μm, respectively, while the expected value would be 48 μm (*i.e.,* the period of M_1_, or equivalently the effective pixel size). These relatively minor discrepancies between experiment and theory may be explained by some degree of shrinking or displacement of the animals inside the cylinders that could not be avoided and led to motion artefacts, which can be expected to have a larger effect in the longer the scans, or by possible system instabilities.

## Discussion

We have presented the first EI XPC micro-CT images of mice, which were acquired to enable a preliminary assessment of the method’s suitability to preclinical imaging. This was motivated by the fact that EI was developed with the aim of fulfilling the criteria for a practicable application in various fields including this one, allowing to build affordable and deployable scanners (by being compatible with laboratory x-ray sources), and achieving relaxed requirements on the system stability (through masks with relatively large periods and apertures). In this study, we have explored the method’s flexibility in implementing different acquisition schemes for balancing the relationship between key image quality metrics (CNR, spatial resolution) and practicality constraints (scan time, dose).

By providing and analysing separate attenuation and phase images, as well as hybrid images in which attenuation and phase contrast are combined, we have shown that the exploitation of phase effects can lead to an improved CNR for soft tissue structures. A quantitative analysis of the muscle-fat interface has revealed a CNR more than three times larger in the phase image than in the attenuation image. The best relative performance (a sixfold increase of CNR over the attenuation image) was observed in one of the hybrid images. Hybrid images have the additional advantage that they do not require that frames are collected at multiple working points. This enables rotating the sample continuously during scans, implying that the total scan time is determined only by the actual exposure time, without contributions from dead times required for repositioning M_1_. This suggests that EI XPC micro-CT scans could be completed within time frames that align with the demands of preclinical imaging. A scan time of 13 min, which we have demonstrated to be feasible, may already be sufficiently short to keep animals under anaesthesia, but scan time could be reduced further if a source with greater power could be used. From a radiation dose point of view, the entrance dose measured in this study (approximately 300 mGy) is within the range of what is delivered by conventional machines [23]); this could however potentially be reduced through the development of new scanning schemes and algorithms that require less data for the reconstruction of high-quality tomographic images. Further, our results reflect that sub-pixel resolutions are currently feasible only when dithering is applied, while it may be possible to replace dithering by “smarter” high-resolution scanning schemes. As dithering involves acquiring multiple frames per rotation angle, this inevitably increases scan time, unless the exposure time per frame is shortened, which could in turn compromise CNR. Therefore, unless alternative scanning schemes become available, dithering should be considered optional, and used, for example, when a specific investigation requires a high level of detail, and scan time and dose delivery are of lesser concern.

Finally, we would like to draw attention to the preliminary nature of the presented work and point to several aspects where we believe that more research is required before EI XPC micro-CT can become a mainstream modality for preclinical imaging. Most pressingly, while the best relationship between CNR and scan time was observed for the hybrid images, the single-frame phase retrieval required to reconstruct these can only accurately retrieve the interface between any two materials in the sample, while the interfaces between other materials are either smoothed (due to an “over-retrieval”), or the refraction-based edge signal is only partially converted into area signal, which might be interpreted as an artefact. In the hybrid images shown, this has led to the blurred appearance of the bones. However, as the simultaneous presence of bones and soft tissue can barely be avoided when imaging small animals, a more general approach to this type of phase retrieval is needed. An appropriate method would be able to reconstruct sharp images for any sample composition, including samples with strong absorbers like bone. First steps have been taken in this regard, *e.g.,* we have developed an extension to the single-frame phase retrieval that can accommodate multiple material interfaces [24]. However, that method requires that the final image is “spliced” from individual reconstructions, which is not straightforward on complex samples. As a second preliminary aspect, this study has not covered dark field imaging, although it was proven by other studies [12, 13] that this contrast channel can provide valuable, complementary information, *e.g.,* on lung tissue. An evaluation of the benefit of using dark field imaging in preclinical studies, based on the EI method, should be pursued as part of future work. Third, the experimental setup used in this study had not been optimised specifically for the purpose of small animal imaging. We are currently carrying out a thorough investigation into the optimal choice of various experimental settings, such as the width of the apertures in M_1_ and M_2_, the total system length and the effect of using different target materials for x-ray generation. We expect that these efforts will lead to an improvement over the results presented in this paper. Finally, the step necessary for realising *in vivo* scans will indeed be to build a system with a gantry that will rotate around a (horizontally positioned) animal bed. Motion artefacts caused by the respiration and heartbeat of a living mouse would be an additional challenge to overcome and would require implementing cardio-respiratory gating, either prospectively or retrospectively [25].

## Conclusion

We believe that our results provide arguments in support of the suitability of EI XPC micro-CT to be applied in preclinical imaging, despite the abovementioned limitations. Therefore, following further development, EI XPC micro-CT could become a solution for improving the visualisation of soft tissue structures in small animals that is both practical and general, removing the need for exogenous contrast agents.
